# Resolutions on the Death of Prof. Charles A. Lee. M. D.

**Published:** 1872-03

**Authors:** Geo. Hadley

**Affiliations:** Sec’y.


### Resolutions on the Death of Prof. Charles A. Lee, M. D.

The Faculty of the Buffalo Medical College, at a recent meeting passed the following resolutions, on the announcement of the death of Prof. Chas. A. Lee:

*Resolved*, That we learn with deep pain and regret the death of our colleague Prof. Charles A. Lee, of Peekskill, N. Y., who has been for so many years our associate in medical teaching, and with whom our relations have always been such as to leave only the kindest remembrances and regrets.

*Resolved*, That we regard the death of one so distinguished for extensive and varied knowledge, so long and well known as one of the venerable dignitaries in medicine as a serious loss to the profession to whose interests he has devoted his life labor.

*Resolved*, That we tender to the family relatives of the deceased our most heartfelt personal sympathy in their bereavement.

*Resolved*, That a copy of these resolutions be sent to the family of the deceased and published in the Buffalo Medical and Surgical Journal.

GEO. HADLEY, Sec'y.

